# Prognostic impact of *ASXL1* mutations in chronic phase chronic myeloid leukemia

**DOI:** 10.1038/s41408-022-00742-1

**Published:** 2022-10-28

**Authors:** Aram Bidikian, Hagop Kantarjian, Elias Jabbour, Nicholas J. Short, Keyur Patel, Farhad Ravandi, Koji Sasaki, Ghayas C. Issa

**Affiliations:** 1 Department of Leukemia, The University of Texas MD Anderson Cancer Center, Houston, TX USA; 2 Department of Hematopathology, The University of Texas MD Anderson Cancer Center, Houston, TX USA

**Keywords:** Chronic myeloid leukaemia, Chronic myeloid leukaemia

## Abstract

While the clinical impact of mutations in the *ABL1* gene on response to therapy in chronic phase chronic myeloid leukemia (CP-CML) is well established, less is known about how other mutations affect prognosis. In a retrospective analysis, we identified 115 patients with CML (71 chronic, 15 accelerated and 29 blast phase) where targeted next-generation sequencing of genes recurrently mutated in myeloid leukemias was performed. *ASXL1* was the most frequently mutated gene in the chronic (14%) and accelerated phase (40%) CML patients, whereas *RUNX1* (20%) was the most common mutation in blast phase. Compared with wild-type *ASXL1*, CP-CML with mutant *ASXL1* was associated with worse event-free survival (EFS) (median of 32.8 vs 88.3 months; *P* = 0.002) and failure-free survival (median of 13.8 vs 57.8 months; *P* = 0.04). In a multivariate analysis, *ASXL1* mutation was the only independent risk factor associated with worse EFS in chronic phase CML with a hazard ratio of 4.25 (95% CI 1.59–11.35, *P* = 0.004). In conclusion, mutations in *ASXL1* are associated with worse outcomes when detected in chronic phase CML.

## Introduction

Tyrosine kinase inhibitors (TKIs) have revolutionized the treatment of chronic myeloid leukemia (CML). However, despite success of this targeted therapy, resistance occurs with *ABL1* kinase domain mutation as the best described mechanism [1]. Approximately 40% of TKI resistance is independent of *BCR::ABL1* signaling [2], and could be mediated by chromosomal instability or possibly mutations in other genes recurrently mutated in myeloid malignancies [3–5].

Driver mutations in non-*ABL1* genes, especially *RUNX1*, are commonly detected in blast phase CML, (BP-CML) [6–10], where there is a strong association between mutational profile and blast phenotype [8]. *ASXL1, BCORL1, RUNX1,* and *TP53* mutations are associated with myeloid phenotype, whereas *CDKN2A/B* and *IKZF1* mutations are more common in the lymphoid blast phase phenotype [8]. Despite the characterization of the mutational profile at blast-phase transformation, less is known about the frequency and clinical impact of non-*ABL1* gene mutations in chronic phase CML (CP-CML). Mutations in *ASXL1* have been detected in CP-CML, though their impact on clinical outcomes is not well-established [11–14]. These mutations could confer suboptimal response to TKIs, but it is unclear whether this would be due to higher co-occurrence with *ABL1* kinase domain mutations [12, 13, 15], or independent epigenetic changes mediated by the polycomb repressive pathways [16]. In addition, other somatic mutations have been detected in CML Philadelphia-negative clones which persisted after optimal TKI response, suggesting a clonal event similar to clonal hematopoiesis of indeterminate potential (CHIP) [17, 18].

Given the unknown clinical impact of these mutations in CP-CML and the lack of clinical guidelines for testing and interpretation [19], we sought to describe the mutational profile of both chronic and advanced-phase CML and study the impact of mutations on TKI response and survival.

## Methods

### Patient selection and mutational analysis

We screened our databases for adult patients diagnosed with CML and identified those where mutational analysis was performed between 2017 and 2022. Targeted next-generation sequencing was done using a panel of 81 genes recurrently mutated in hematologic malignancies (Supplemental Table 1) [20]. To ensure the accuracy of *ASXL1* p.G646fs mutation detection, we used a combination of sequencing chemistry, sequencing platform, internal VAF database, and a stringent VAF cut-off (>10%) to avoid sequencing artifacts across this homopolymer region [21]. *ABL1* kinase domain mutations were identified using a previously described nested PCR-based cDNA sequencing assay, targeting codons 221 to 500, with an additional pyrosequencing step for T315I mutation detection [22]. Accelerated phase CML (AP-CML) and BP-CML were defined as per MD Anderson Cancer Center (MDACC) criteria [23]. A total of 115 evaluable patients were identified, among whom 71 had CP-CML (41 tested at diagnosis), 15 had AP-CML and 29 had BP-CML (Supplemental Fig. 1). This study was approved by the institutional review board and performed in accordance with the Declaration of Helsinki.

### Response and outcome definitions

Response and survival outcomes were determined as previously described [24]. Major molecular response (MMR) was defined as a *BCR::ABL1/ABL1* ratio of ≤0.1% on the international scale (IS). Molecular response with a 4-log reduction (MR4) and molecular response with a 4.5-log reduction (MR4.5) were defined as *a BCR::ABL1/ABL1* ratio of ≤0.01% and ≤0.0032% (IS), respectively. Event-free survival (EFS) was measured from the start of treatment with first-line TKI to the date of any of the following events: loss of complete hematologic remission, loss of major cytogenetic response (MCyR), failure to achieve MCyR by 12 months, progression to accelerated or blast phase, or death from any cause. Failure-free survival (FFS) was measured similarly to EFS with the addition of treatment discontinuation due to resistance or intolerance as an event [25]. Overall survival (OS) was measured from the time of treatment with first-line TKI to time of death from any cause as an event.

### Statistical analysis

Patient characteristics were summarized using median (range) for continuous and frequency (percentage) for categorical variables. Fisher’s exact test and Wilcoxon rank-sum test were used to assess differences in categorical and continuous variables, respectively. Survival probabilities were estimated by the Kaplan–Meier method and the log-rank test was used for comparisons. Univariate and multivariate analyses were used to assess the association between patient characteristics and survival outcomes.

## Results

### Baseline characteristics

Table 1 summarizes the baseline characteristics and treatments of patients with CP-CML by mutational status. The rate of *ABL1* mutations was higher in patients with mutant *ASXL1* compared to patients with no mutations, albeit not statistically significant (29% vs 11%, *P* = 0.3).

**Table 1:** Baseline characteristics.

Characteristic	*ASXL1* mutation (*N* = 10)	*P*	Other non-*ABL1* mutation (*N* = 13)	*P*	No mutation (*N* = 48)
Age, median (range)	62 (27–73)	0.7	60 (25–80)	0.9	59 (18–77)
Female, no. (%)	5 (50%)	0.9	5 (43%)	0.9	22 (46%)
WBC, median × 10^9^ (range)	42.7 (3–281)	0.2	30 (3–341)	0.4	84.8 (3–538)
Hb, median g/dL (range)	10.2 (8.0–13.9)	0.2	11.6 (7.5–15.5)	0.9	12.0 (6.7–16.3)
Platelets, median × 10^9^ (range)	405 (137–1265)	0.4	193 (74–375)	0.02	333 (19–1832)
Basophils, median% (range)	2 (0–5)	0.8	1 (0–4)	0.03	2 (0–5)
BM Blasts, median% (range)	2 (0–5)	0.6	1 (0–6)	0.9	2 (0–5)
*ABL1* mutation, no./tested (%)	2/7 (29%)	0.3	1/9 (11%)	0.9	2/18 (11%)
Sokal Score, no./tested (%)		0.5		0.3	
Low	1/8 (12%)		3/10 (30%)		9/39 (23%)
Intermediate	4/8 (50%)		7/10 (70%)		23/39 (59%)
High	3/8 (38%)		0/10 (0%)		7/39 (18%)
1st Line Therapy		0.5		0.2	
Imatinib	2 (20%)		4 (31%)		8 (17%)
Dasatinib	7 (70%)		3 (23%)		27 (56%)
Other	1 (10%)		6 (46%)		13 (27%)
HSCT	2 (20%)	0.02	1 (8%)	0.3	1 (2%)

*HSCT* hematopoietic stem cell transplant.

In BP-CML, the median bone marrow blast percentage at presentation was higher in the mutation group (45%) compared with the no mutation group (24%) albeit not statically significant (*P* = 0.07). The baseline characteristics for AP-CML and blast BP-CML are summarized in Supplemental Tables 2 and 3.

### Mutational profile in CP-CML

**Fig. 1:**
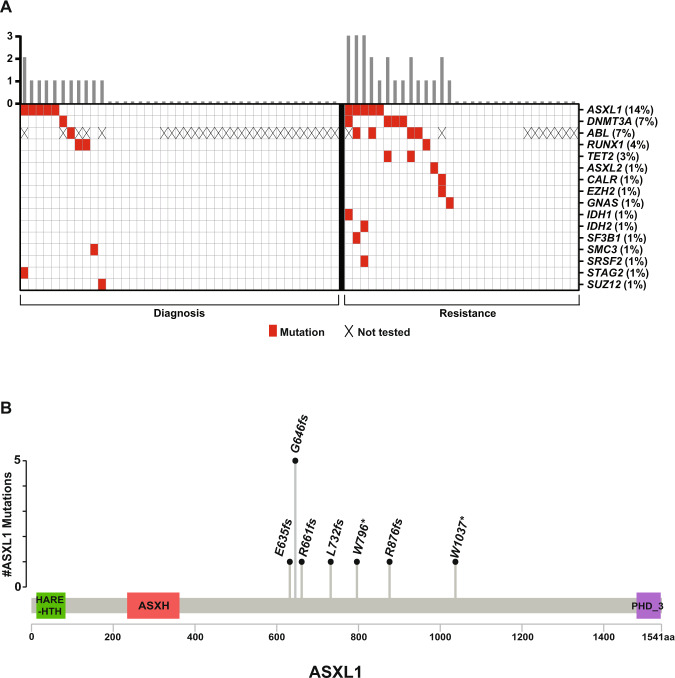
Mutational profile of chronic phase CML (CP-CML) patients. **A** Distribution of mutations in patients with CP-CML. **B** Frequency of various *ASXL1* mutations identified. aa amino acids.

Among the 71 evaluable patients, 23 (32%) had at least one non-*ABL1* mutation with 6 (8%) carrying two or more non-*ABL1* mutations (Fig. 1A). The prevalence of mutations was lower in patients who were tested at diagnosis (10 out of 41) compared with patients who were tested at resistance or at loss of response (13 out of 30) (24% vs 43%; *P* = 0.1) The most common mutation was *ASXL1* detected in 10 (14%) of all tested patients followed by *DNMT3A* in 5 (7%) and *RUNX1* in 3 (4%) patients. Mutations in 12 other genes were detected at least once (Fig. 1A).

Seven different *ASXL1* mutations were detected among 10 patients, with one patient carrying two concomitant *ASXL1* mutations. These were either frameshift or nonsense mutations in exon 13 that resulted in a truncated ASXL1 protein. The most common mutation was G646fs, detected in five patients (Fig. 1B).

### Response rates and time to response

Cytogenetic and molecular response rates as well as times to attaining these responses (TTR) are highlighted in Table 2 and Supplemental tables 4 and 5. One of the patients in the mutation group was lost to follow-up soon after treatment initiation and therefore was not included in response analyses. A trend of lower early response rates (33% vs 61%, *P* = 0.2) and longer time to MMR (17.5 vs 9.0 months, *P* = 0.2), MR4 (20.7 vs 16.2 months, *P* = 0.4) and MR4.5 (48.7 vs 23.0 months, *P* = 0.4) were observed in patients with mutant *ASXL1*, compared to wild-type counterpart, albeit not statistically significant (Supplemental Table 5).

**Table 2:** Responses.

Response	*ASXL1* (*N* = 9)			Other mutations (*N* = 13)			No Mutation (*N* = 48)	
	N (%)	Median TTR^a^ (range)	*P*	*N* (%)	Median TTR^a^ (range)	*P*	*N* (%)	Median TTR^a^ (range)
MCyR	9 (100%)	3.3 (1–65)	0.9	12 (92%)	3.7 (1–13)	0.5	45 (94%)	3.4 (1–52)
CCyR	8 (89%)	9.7 (3–66)	0.4	11 (85%)	6.0 (3–13)	0.2	45 (94%)	6.0 (2–31)
MMR	7 (78%)	17.5 (5–66)	0.7	10 (77%)	6.7 (3–70)	0.7	39 (81%)	9.2 (3–142)
MR4	5 (56%)	29.1 (10–86)	0.5	8 (62%)	15.5 (4–76)	0.7	32 (67%)	16.3 (3–167)
MR4.5	4 (44%)	48.7 (11–89)	0.7	8 (62%)	27.0 (12–120)	0.7	26 (54%)	17.2 (4–73)
Early response^b^	3/9 (33%)	–	0.3	5/12 (42%)	–	0.2	28/42 (67%)	–

Early response is reported as number/evaluable.

*TTR* time to response in months, *MCyR* major cytogenetic response, *CCyR* complete cytogenetic response, *MMR* major molecular response, *MR4* molecular response with a 4-log reduction, *MR4.5* molecular response with a 4.5-log reduction.

^a^Wilcoxon rank sum test was performed to assess the difference in TTR (*P < 0.05).

^b^Defined as *BCR::ABL1* < 10% at 3 months.

### Impact on survival

**Fig. 2:**
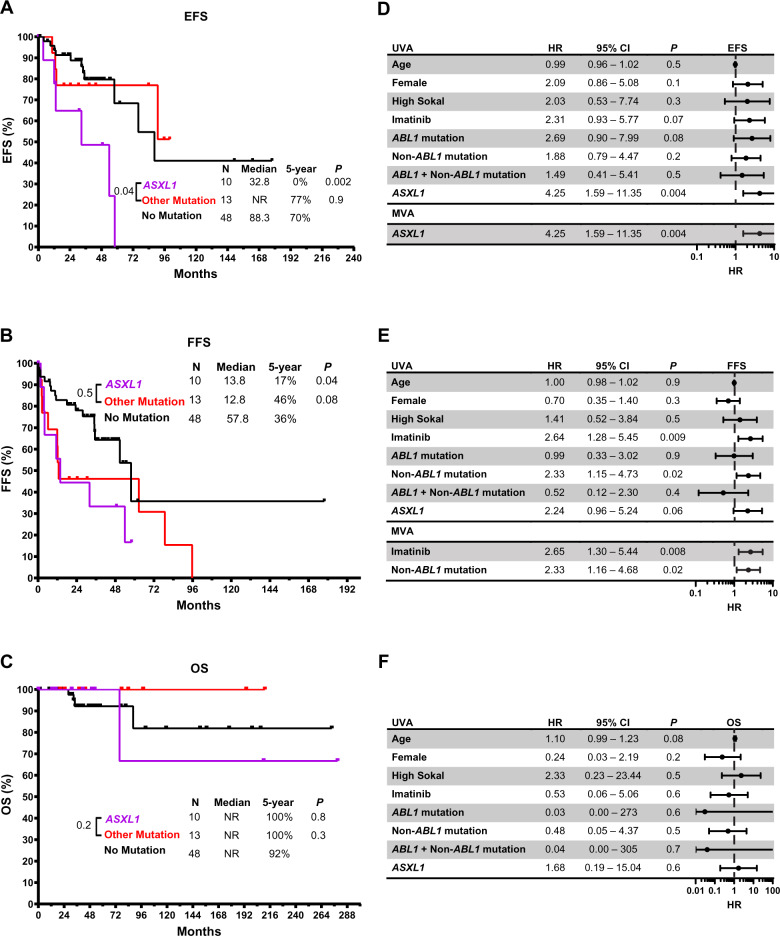
Impact of *ASXL1* mutations on survival in patients with CP-CML. **A** Event-free survival (EFS). **B** Failure-free survival (FFS) and **C** overall survival (OS) comparing patients with mutations in *ASXL1* vs mutations in other genes or no mutation. Univariate (UVA) and multivariate (MVA) analyses of factors predicting: **D** EFS, **E** FFS, and **F** OS. m months.

Patients who had at least one non-*ABL1* mutation had worse FFS compared to those with no mutations (Median FFS 13.3 months vs 57.8 months; *P* = 0.02). There was no statistically significant difference in EFS or OS comparing the two groups (Supplemental Fig. 2). Compared with patients with no mutations, patients who had *ASXL1* mutations had worse EFS (median of 32.8 months vs 88.3 months; *P* = 0.002) (Fig. 2A, Supplemental Fig. 3B) and worse FFS (median of 13.8 months vs 57.8 months; *P* = 0.04) (Fig. 2B). EFS and FFS of patients with mutations other than *ASXL1* were not significantly different from those with no mutations. There was no significant difference in OS in our cohort based on the mutational profile of the patients (Fig. 2C), albeit a trend of worse OS in patients with mutated *ASXL1* when considering only CML-related mortality (Supplemental Fig. 3C).

Among patients who were tested for mutations at diagnosis, those who had *ASXL1* mutations had significantly worse EFS (median of 30.3 months vs not reached; *P* = 0.02) and FFS (median of 12.6 months vs not reached; *P* = 0.02) when compared to patients with no mutations (Supplemental figure 4). In patients tested at loss of response, there was still a trend of worse EFS in patients with *ASXL1* mutation, albeit not statistically significant (Supplemental Fig. 5).

### Multivariate analysis

In order to assess the impact of confounding variables on survival, we conducted univariate and multivariate analyses to predict the determinants of EFS, FFS, and OS. *ASXL1* mutation was identified as an independent risk factor associated with worse EFS with a hazard ratio (HR) of 4.25 (95% CI 1.59–11.35, *P* = 0.004) (Fig. 2D). On the other hand, having any non-*ABL1* mutation (HR of 2.33, 95% CI 1.16–4.68, *P* = 0.02) and treatment with imatinib (HR of 2.65, 95% CI 1.30–5.44, *P* = 0.008) were each independent risk factors associated with worse FFS (Fig. 2E).

### Advanced phase CML

**Fig. 3:**
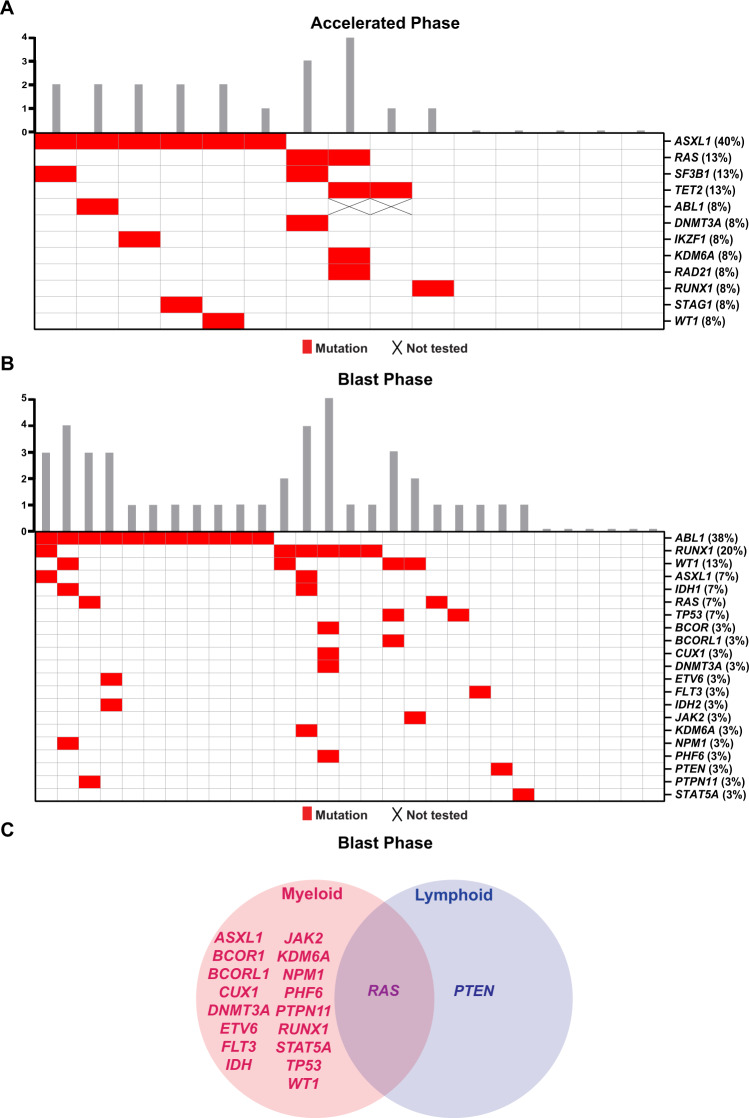
Mutational profile of accelerated phase (AP-CML) and blast phase (BP-CML) CML. **A** Distribution of mutations in patients with AP-CML, and **B** BP-CML. **C** Association between non-*ABL1* gene mutations and the blast phase phenotype.

Fifteen patients with AP-CML and 29 patients with BP-CML had mutational analysis performed (Supplemental Tables 2 and 3). Among the 15 patients with AP-CML, 10 (67%) had at least one non-*ABL1* mutation (Fig. 3A). The most common mutation was *ASXL1* detected in 6 (40%) patients followed by *RAS, SF3B1,* and *TET2* detected in 2 (13%) patients each. Mutations were also detected in 7 other non-*ABL1* genes as highlighted in (Fig. 3A).

*ABL1* was the most common mutation in BP-CML, detected in 11 (38%) patients. Sixteen (56%) patients had at least one non-*ABL1* mutation and 9 (31%) had two or more mutations (Fig. 3B). *RUNX1* was the most frequently mutated gene in 6 (21%) patients, followed by *WT1* in 4 (13%) and *ASXL1* in 2 (7%) patients. *PTEN* mutation was detected in one patient who had lymphoid BP-CML and *RAS* mutation was detected in both lymphoid and myeloid phenotypes (one patient each). All the other gene mutations were associated with myeloid blast phenotype (Fig. 3C).

Among the 29 BP-CML patients, 26 were followed for a median of 18.1 months. Patients who had at least one non-*ABL1* mutation had significantly worse EFS (1-year survival of 17% vs 61%, *P* = 0.007) (Supplemental figure 6). Multivariate analysis revealed that having at least one non-*ABL1* mutation was an independent risk factor associated with worse EFS (HR = 5.42, 95% CI 1.2–23.8, *P* = 0.03) (Supplemental Table 6). Mutation status did not affect OS or FFS, whereas a myeloid phenotype was associated with worse OS (HR = 5.67, 95% CI 1.3–25.6, *P* = 0.02) (Supplemental Tables 7 and 8).

### AML development

When screening our databases for patients for this study, we also identified patients who had history of CML and later developed de novo myelodysplastic syndrome (MDS) or acute myeloid leukemia (AML), with low *BCR::ABL1* levels (<1% IS). Interestingly, one of these patients with *ASXL1, IDH2* and *SRSF2* mutations identified in CP-CML, developed *de novo* AML 6 years after his initial CML diagnosis. Mutational analysis at the time of AML diagnosis revealed the same mutations.

## Discussion

In this study we report on the mutational profile of CML patients who were tested in the clinical setting. Mutations in *ASXL1* were the most common alteration in CP-CML, detected in 14% of all evaluable patients and in 12% of patients tested at diagnosis. These mutations were associated with significantly worse EFS and FFS.

The presence of non-*ABL1* mutations and their potential adverse prognostic impact has been previously described [14]; however, to our knowledge, our study represents the first report on the impact of *ASXL1* mutations on survival, adding to recently emerging evidence of suboptimal TKI response associated with these mutations [15]. There was no difference in OS when these mutations were detected, which could be due to the relatively short follow-up period (median follow-up of 42 months), or the excellent outcomes of CP-CML with the current standard of care. Mutations in *RUNX1* were the most common non-*ABL1* mutations among patients with BP-CML, mostly among those with a myeloid blast phenotype, an association reported in previous studies [8]. While none of the CP-CML patients in our cohort progressed to AP or BP, a small fraction with CP-CML developed *de novo* MDS or AML, including one patient who had the same mutational profile during his CML treatment. This could be explained by the presence of non-*ABL1* mutations in Philadelphia-negative clones [17, 18]. Our findings could be biased by the fact that only 41 of 71 (58%) patients were tested for mutations at diagnosis, whereas 30 (42%) were tested only when clinicians suspect resistance or suboptimal response, therefore confounding our predicted associated risks with adverse outcomes. Prospective analysis with unbiased testing of these mutations could better determine their associated prognostic impact.

Although larger studies are needed, our findings support the role of broad mutational analysis in CP-CML, especially those with suboptimal response to therapy and absence of *ABL1* mutations.

## Supplementary information

Supplemental material

## Data Availability

Data generated and/or analyzed during the current study are available from the corresponding author on reasonable request.
