# Supplementary material for: Prognostic impact of ASXL1 mutations in chronic phase chronic myeloid leukemia
Source: Blood Cancer J. 2022 Oct 28;12(10):144. doi: 10.1038/s41408-022-00742-1 (PMC9616867; doi:10.1038/s41408-022-00742-1)

Supplemental Table 1: Genes, exons and codons tested by 81-gene targeted next-generation sequencing panel.

Gene	Exons (Codons) Tested
***ANKRD26* (NM_014915)**	1 (1-6)
***ASXL1* (NM_015338)**	11-12 (362-1542)
***ASXL2* (NM_018263)**	11-12 (381-1436)
***BCOR* (NM_017745)**	2-8 (1-1249), 9-15 (1264-1644), 15 (1663-1722)
***BCORL1* (NM_021946)**	1-6 (1-1261), 6 (1292-1323), 6-12 (1326-1712)
***BRAF* (NM_004333)**	11 (439-478), 15 (581-620)
***BRINP3* (NM_199051)**	2-8 (1-471), 8 (475-767)
***CALR* (NM_004343)**	9 (352-418)
***CBL* (NM_005188)**	7-9 (336-477)
***CBLB* (NM_170662)**	7-10 (282-469)
***CBLC* (NM_012116)**	7-9 (336-454), 10 (465-475)
***CEBPA* (NM_004364)**	1 (1-96), 1 (146-175), 1 (178-201), 1 (215-244), 1 (275-358)
***CREBBP* (NM_004380)**	2-8 (29-608), 9-31 (615-1943), 31 (1950-2443)
***CRLF2* (NM_022148.2)**	6 (217-256)
***CSF3R* (NM_156039)**	14 (575-622), 17 (681-864)
***CUX1* (NM_181552)**	2-6 (11-172), 6-14 (174-408)
***DDX41* (NM_016222)**	1-17 (1-623)
***DNMT3A* (NM_022552)**	8-22 (286-862), 23 (866-913)
***EED* (NM_003797)**	1-2 (1-69), 2-8 (71-287), 9-12 (289-442)
***ELANE* (NM_001972)**	1-2 (5-48), 2 (69-75), 4-5 (123-268)
***ETNK1* (NM_018638)**	3 (228-275)
***ETV6* (NM_001987)**	1-8 (1-453)
***EZH2* (NM_004456)**	2-5 (1-158), 5-6 (160-205), 7 (209-217), 8-19 (243-732), 20 (752)
***FBXW7* (NM_033632)**	9-12 (413-708)
***FLT3* (NM_004119)**	11-20 (437-847)
***GATA1* (NM_002049)**	2-3 (1-84)
***GATA2* (NM_032638)**	2-5 (1-377), 5-6 (379-481)
***GFI1* (NM_005263)**	2 (2-39)
***GNAS* (NM_000516)**	8 (200-202), 11 (315-324)
***HNRNPK* (NM_002140)**	3-17 (1-465)
***HRAS* (NM_005343)**	2-3 (1-70), 3-4 (74-150)
***IDH1* (NM_005896)**	4 (132-133)
***IDH2* (NM_002168)**	4 (125-178)
***IKZF1* (NM_006060)**	2-8 (1-443), 8 (445-518)
***IL2RG* (NM_000206)**	1-8 (1-340), 8 (352-370)
***IL7R* (NM_002185)**	5-7 (180-292)
***JAK1* (NM_002227)**	3-22 (3-1023), 22-24 (1026-1123)
***JAK2* (NM_004972)**	10 (405-442), 12-14 (505-622), 16 (665-711), 18 (762-812)
***JAK3* (NM_000215)**	2-23 (1-1069)
***KDM6A* (NM_021140)**	1-29 (1-1402)
***KIT* (NM_000222)**	8-9 (411-514), 11 (550-592), 17 (788-828)

***KMT2A* (NM_005933)**	2-4 (145-1075), 4-13 (1081-1561), 14-15 (1566-1665), 27 (2166-2175), 27 (2178-2362), 27 (2365-3216), 27 (3219-3327), 27 (3331-3582)
***KRAS* (NM_004985)**	2-4 (1-150)
***MAP2K1* (NM_002755)**	2 (27-90), 3 (98-146)
***MPL* (NM_005373)**	10 (490-522), 12 (552-636)
***NF1* (NM_001042492)**	2-5 (21-189), 6-7 (201-226), 7-13 (232-468), 13-14 (478-547), 15-26 (568-1146), 26-31 (1160-1378), 31-35 (1380-1550), 35-38 (1563-1868), 39 (1870-1884), 39-47 (1886-2322), 47-51 (2325-2491), 51-52 (2494-2555), 52-58 (2568-2840)
***NOTCH1* (NM_017617)**	26-28 (1529-1795), 34 (2069-2230), 34 (2234-2286), 34 (2290-2309), 34 (2290-2556), 34 (2069-2230), 34 (2234-2286), 34 (2309-2556)
***NPM1* (NM_002520)**	11 (283-295)
***NRAS* (NM_002524)**	2-4 (1-150)
***PAX5* (NM_016734)**	1-10 (1-392)
***PHF6* (NM_032458)**	2-10 (1-366)
***PIGA* (NM_002641)**	2 (1-6), 2-6 (13-485)
***PML* (NM_033238)**	3 (201-255)
***PRPF40B* (NM_001031698)**	2-19 (2-609), 19-20 (611-658), 20-26 (668-893)
***PTEN* (NM_000314)**	7-8 (212-342)
***PTPN11* (NM_002834)**	3-4 (46-125), 7 (253-285), 12 (460-462), 12-13 (465-533)
***RAD21* (NM_006265)**	2-14 (1-632)
***RARA* (NM_000964)**	6-7 (211-338)
***RUNX1* (NM_001754)**	2-9 (1-437), 9 (456-474)
***SETBP1* (NM_015559)**	4 (838-885)
***SF1* (NM_004630)**	1-12 (1-524), 13 (528-578), 13 (607-640)
***SF3A1* (NM_005877)**	1-9 (1-424), 9-16 (427-794)
***SF3B1* (NM_012433)**	13-16 (574-790)
***SH2B3* (NM_005475)**	2 (1-118), 2 (132-164), 2 (212-226), 2-8 (233-576)
***SMC1A* (NM_006306)**	1-25 (13-1234)
***SMC3* (NM_005445)**	1-6 (1-110), 6-16 (113-504), 16-17 (507-580), 17-29 (591-1217)
***SRSF2* (NM_003016)**	1 (1-38), 1 (45-121)
***STAG1* (NM_005862)**	2 (1-5), 3-22 (10-738), 22-27 (740-953), 27-34 (955-1259)
***STAG2* (NM_006603)**	2-33 (1-1232)
***STAT3* (NM_139276)**	17 (489-509), 17-22 (521-715)
***STAT5A* (NM_003152)**	3-7 (1-214), 8-20 (249-795)
***STAT5B* (NM_012448)**	16 (636-693)
***SUZ12* (NM_015355)**	1 (21-84), 2-3 (92-116), 4-5 (129-169), 6-16 (174-740)
***TERC* (NR_001566)**	1 (1-36)
***TERT* (NM_198253)**	1 (1-24), 1 (66), 2 (74-165), 2 (246-300), 2 (312-342), 2-4 (349-630), 4-5 (633-692), 6-16 (711-1133)
***TET2* (NM_001127208)**	3 (1-77), 3 (91-826), 3 (829-853), 3-11 (867-2003)
***TP53* (NM_000546)**	2 (1-25), 4-11 (80-394)
***U2AF1* (NM_006758)**	2 (15-44), 6 (117-161)
***U2AF2* (NM_007279)**	1 (1-17), 3-5 (62-161), 6-12 (163-473)
***WT1* (NM_024426)**	1 (1-5), 1 (7-63), 1 (77-96), 1-10 (127-518)
***ZRSR2* (NM_005089)**	1-4 (1-90), 5-11 (105-483)

Supplemental Table 2: Baseline characteristics of patients with accelerated phase CML (AP-CML) who underwent mutational analysis.

Characteristic	Mutation (N=10)	No Mutation (N=5)	*P*
**Age, median (range)**	61 (32 – 89)	62 (37 – 79)	0.8
**Female, no. (%)**	4 (40%)	2 (40%)	0.9
**De novo AP-CML**	6 (60%)	1 (20%)	0.3
**WBC, median x 10^9 (range)**	13.3 (3 – 126)	66.1 (7 – 411)	0.1
**Hb, median g/dL (range)**	9.8 (7.2 – 13.4)	11.9 (6.7 – 17.9)	0.6
**Platelets, median x 10^9 (range)**	131 (19 – 738)	197 (148 – 600)	0.4
**Basophils, median% (range)**	2 (0 – 58)	2 (1 – 9)	0.8
**BM Blasts, median% (range)**	10 (1 – 17)	3 (0 – 5)	0.1
***ABL1* mutation, no./tested (%)**	1/6 (17%)	0/5 (0%)	0.9
**1st line therapy**			
**Dasatinib**	3 (30%)	2 (40%)	0.9
**Bosutinib + azacytidine**	1 (10%)	1 (20%)	0.9
**Other**	6 (60%)	2 (40%)	0.6
**Prior therapy lines, median (range)**	2 (0 – 9)	2 (0 – 4)	0.9
**Stem cell transplant**	1 (10%)	0 (0%)	0.9

WBC, white blood cells; Hb, hemoglobin; BM, bone marrow;

Supplemental Table 3: Baseline characteristics of patients with blast phase CML (BP-CML) who underwent mutational analysis.

Characteristic	Mutation (N=16)	No Mutation (N=13)	*P*
**Age, median (range)**	56 (26 – 82)	62 (37 – 79)	0.8
**Female, no. (%)**	8 (50%)	4 (31%)	0.5
**De novo BP-CML**	5 (31%)	6 (46%)	0.5
**WBC, median x 10^9 (range)**	22.1 (2 – 164)	66.1 (7 – 411)	0.1
**Hb, median g/dL (range)**	9.7 (6.4 – 14.5)	9.8 (7.0 – 10.8)	0.4
**Platelets, median x 10^9 (range)**	70 (19 – 1032)	68 (22 – 1612)	0.7
**Basophils, median% (range)**	0 (0 – 5)	0 (0 – 17)	0.1
**BM Blasts, median% (range)**	45 (2 – 99)	24 (1 – 90)	0.07
**Myeloid phenotype, no. (%)**	14 (88%)	6 (46%)	0.04
***ABL1* mutation (%)**	4 (25%)	7 (54%)	0.1
**1st line therapy**			
**TKI + chemotherapy**	1/13 (7%)	5/13 (39%)	0.2
**TKI alone**	4/13 (31%)	3/13 (23%)	0.9
**Chemotherapy alone**	4/13 (31%)	1/13 (7%)	0.3
**TKI + other combination**	4/13 (31%)	4/13 (31%)	0.9
**Prior therapy lines, median (range)**	2 (0 – 9)	1 (0 – 4)	0.7
**Stem cell transplant**	1 (10%)	0 (0%)	0.9

WBC, white blood cells; Hb, hemoglobin; BM, bone marrow; TKI, tyrosine kinase inhibitor
1st line therapy reported as values/evaluable (%)

Supplemental Table 4: Response rates of patients with chronic phase CML (CP-CML) carrying at least one non-*ABL1* mutation (Mutation) vs no mutation

Response	Mutation (N=22)			No Mutation (N=48)	
	N (%)	Median TTR[1] (range)	*P*	N (%)	Median TTR[1] (range)
**MCyR**	21 (95%)	3.3 (1 – 65)	0.9	45 (94%)	3.4 (1 – 52)
**CCyR**	19 (86%)	6.2 (3 – 66)	0.6	45 (94%)	6.0 (2 – 31)
**MMR**	17 (77%)	9.1 (3 – 70)	0.8	39 (81%)	9.2 (3 – 142)
**MR4**	13 (59%)	18.7 (4 – 86)	0.6	32 (67%)	16.3 (3 – 167)
**MR4.5**	12 (55%)	25.5 (4 – 120)	0.9	26 (54%)	17.2 (4 – 73)
**Early Response[2]**	8/21 (38%)	-	0.06	28/42 (67%)	-

TTR, Time to response in months
MCyR, Major cytogenetic response; CCyR, Complete cytogenetic response; MMR, Major molecular response; MR4, molecular response with a 4-log reduction; MR4.5, molecular response with a 4.5-log reduction

[1]Wilcoxon rank sum test was performed to assess the difference in TTR (* *P*<0.05)
[2]Defined as BCR-ABL <10% at 3 months
Early Response reported as number/evaluable

Supplemental Table 5: Response rates and time to response by *ASXL1* mutational status.

Response	*ASXL1* (N=9)		No/Other Mutations (N=61)			
	N (%)	Median TTR (range)	N (%)	Median TTR (range)	*P* (RR)	*P*[1] (TTR)
**MCyR**	9 (100%)	3.3 (1–65)	57 (93%)	3.1 (1–52)	0.9	0.8
**CCyR**	8 (89%)	9.7 (3–66)	56 (92%)	6.0 (2–31)	0.6	0.3
**MMR**	7 (78%)	17.5 (5–66)	49 (80%)	9.0 (3–142)	0.9	0.2
**MR4**	5 (56%)	20.7 (10–86)	40 (66%)	16.2 (3–167)	0.7	0.4
**MR4.5**	4 (44%)	48.7 (11–89)	34 (56%)	23.0 (4–120)	0.7	0.4
**Early Response[2]**	3/9 (33%)	-	33/54 (61%)	-	0.2	0.2

TTR, Time to response in months
MCyR, Major cytogenetic response; CCyR, Complete cytogenetic response; MMR, Major molecular response; MR4, molecular response with a 4-log reduction; MR4.5, molecular response with a 4.5-log reduction
[1]Wilcoxon rank-sum analysis was performed to assess the difference in TTR
[2]Defined as *BCR::ABL1* <10% at 3 month
Early response is reported as number/evaluable

Supplemental Table 6: Univariate (UVA) and Multivariate (MVA) analyses of factors predicting event-free survival (EFS) in blast phase CML (BP-CML).

UVA	HR	95% CI	*P*
**Age**	0.95	(0.92 – 0.98)	0.005
**Female**	1.32	(0.51 – 3.40)	0.6
**Chemotherapy alone**	7.43	(2.29 – 24.10)	0.001
**Stem cell transplant**	0.98	(0.34 – 2.80)	0.9
**Myeloid phenotype**	4.43	(1.41 – 13.93)	0.01
***ABL1* mutation**	1.15	(0.45 – 2.90)	0.8
**Non-*ABL1* mutation**	3.73	(1.35 – 10.34)	0.01
***ABL1* + non-*ABL1* mutation**	3.26	(1.03 – 10.25)	0.04
RUNX1	1.55	(0.44 – 5.48)	0.5
WT1	3.76	(1.15 – 12.28)	0.03
ASXL1	3.60	(0.76 – 17.05)	0.1
**MVA**			
**Age**	0.96	(0.91 – 1.01)	0.1
**Chemotherapy alone**	2.55	(0.37 – 17.60)	0.3
**Myeloid phenotype**	1.59	(0.40 – 6.36)	0.5
**Non-*ABL1* mutation**	5.42	(1.23 – 23.82)	0.03
***ABL1* + non-*ABL1* mutation**	0.60	(0.15 – 2.44)	0.5
WT1	1.06	(0.23 – 4.84)	0.9

Supplemental Table 7: Univariate (UVA) and Multivariate (MVA) analyses of factors predicting failure-free survival (FFS) in blast phase CML (BP-CML).

UVA	HR	95% CI	*P*
**Age**	0.98	(0.95 – 1.01)	0.9
**Female**	0.77	(0.32 – 1.83)	0.5
**Chemotherapy alone**	2.23	(0.78 – 6.39)	0.1
**Stem cell transplant**	1.82	(0.69 – 4.82)	0.2
**Myeloid phenotype**	1.77	(0.70 – 4.45)	0.2
***ABL1* mutation**	1.49	(0.63 – 3.53)	0.4
**Non-*ABL1* mutation**	1.35	(0.57 – 3.20)	0.5
***ABL1* + non-*ABL1* mutation**	1.14	(0.38 – 3.41)	0.8
RUNX1	1.14	(0.38 – 3.45)	0.8
WT1	2.64	(0.72 – 9.70)	0.1
ASXL1	3.08	(0.65 – 14.58)	0.2

Supplemental Table 8: Univariate (UVA) and Multivariate (MVA) analyses of factors predicting overall survival (OS) in blast phase CML (BP-CML).

UVA	HR	95% CI	*P*
**Age**	0.99	(0.95 – 1.02)	0.5
**Female**	1.27	(0.44 – 3.64)	0.7
**Chemotherapy alone**	1.85	(0.49 – 7.03)	0.4
**Stem cell transplant**	1.12	(0.38 – 3.31)	0.8
**Myeloid phenotype**	5.67	(1.26 – 25.55)	0.02
***ABL1* mutation**	1.18	(0.41 – 3.40)	0.8
**Non-*ABL1* mutation**	2.05	(0.68 – 6.21)	0.2
***ABL1* + non-*ABL1* mutation**	3.14	(0.94 – 10.47)	0.06
RUNX1	0.88	(0.20 – 3.97)	0.9
WT1	3.97	(0.97 – 16.26)	0.06
ASXL1	1.07	(0.14 – 8.23)	0.9
**MVA**			
**Myeloid Phenotype**	5.67	(1.26 – 25.55)	0.02

Supplemental Figure 1: Patient selection

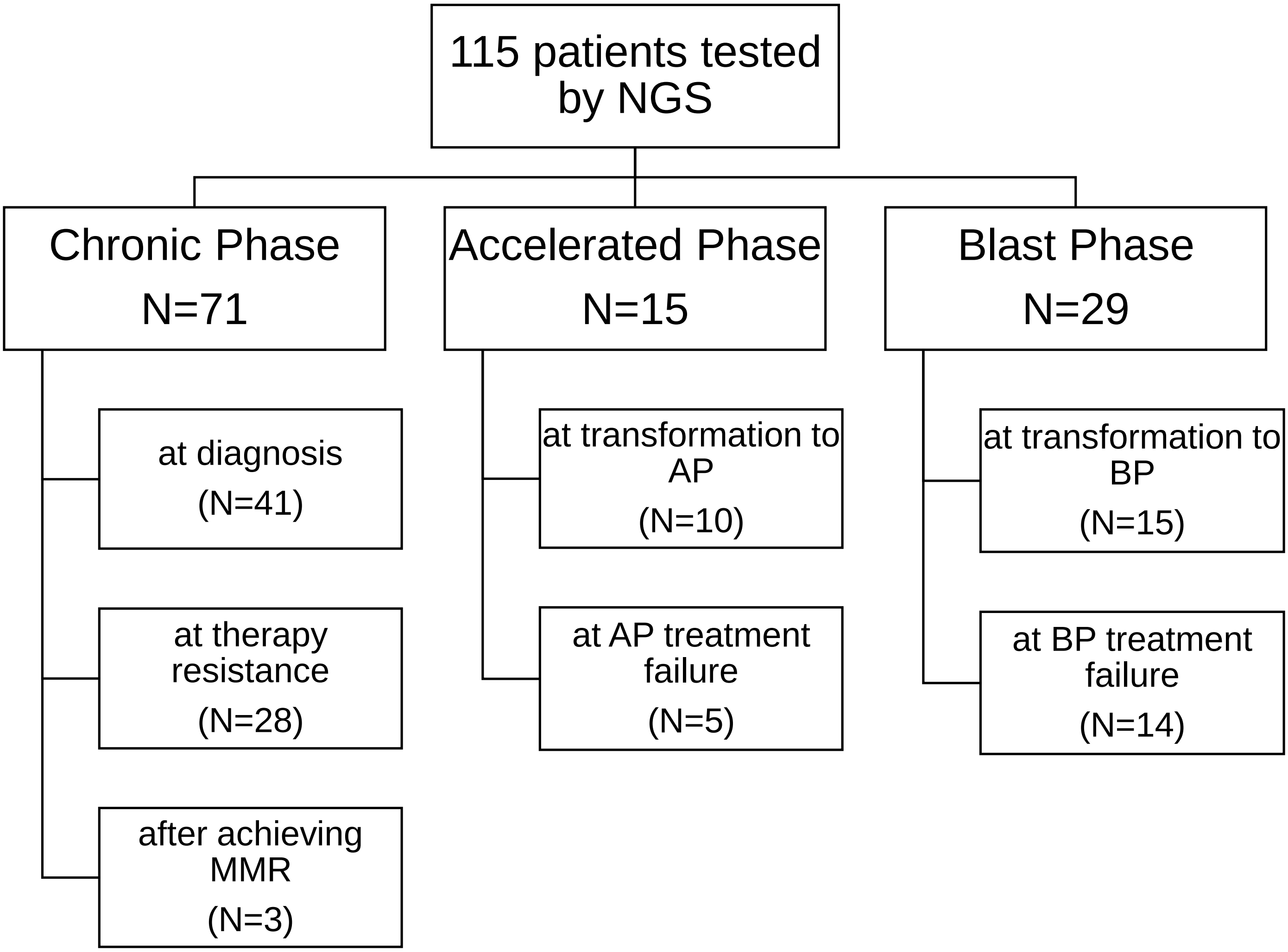

Supplemental Figure 2: Impact of non-*ABL1* gene mutations on survival in chronic phase CML.
A. Event-free survival (EFS) **B.** Failure-free survival (FFS) **C.** Overall survival (OS)

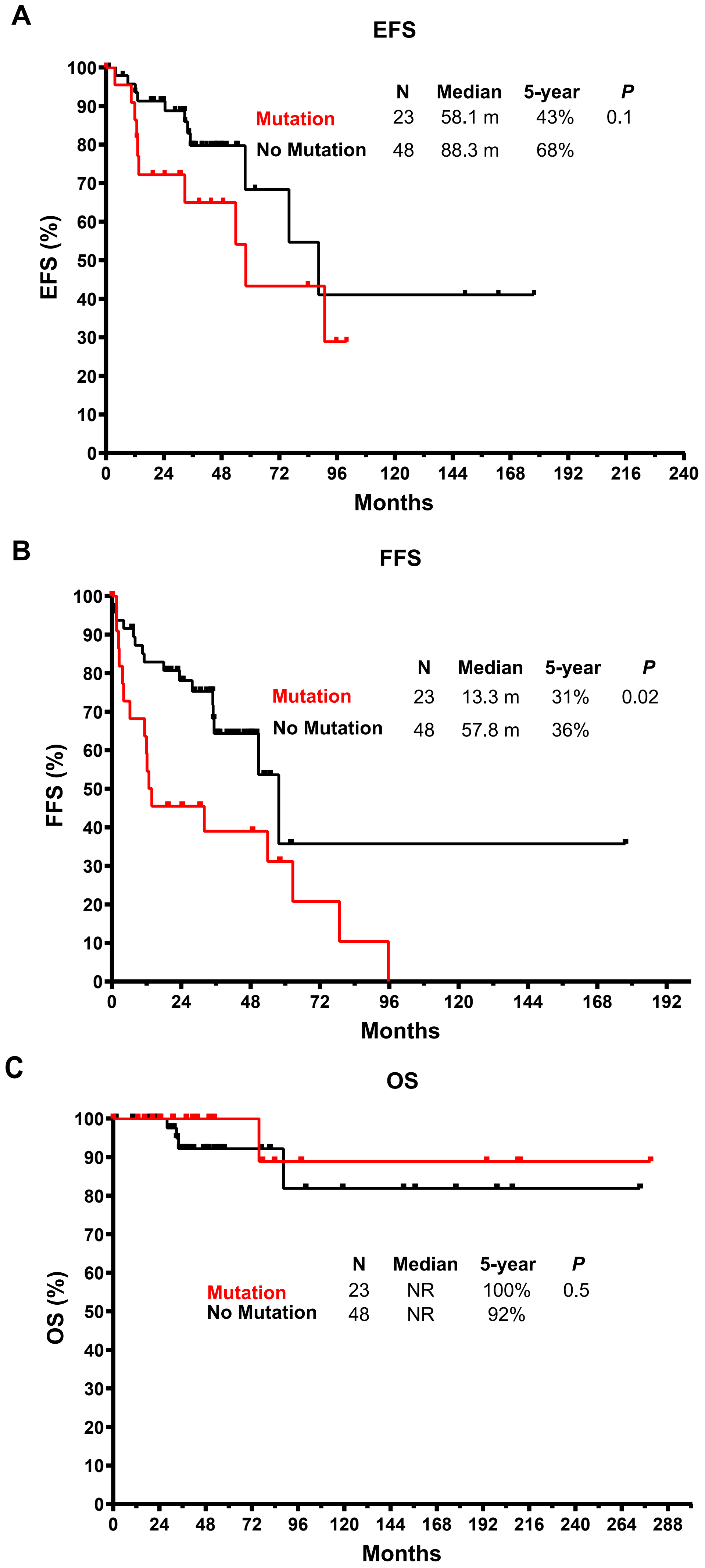

Supplemental Figure 3: Impact of *ASXL1* mutations on survival in patients with chronic phase CML, censored for death due to CML-unrelated causes. A. Event-free survival (EFS) **B.** Failure-free survival (FFS) and **C.** Overall survival (OS).

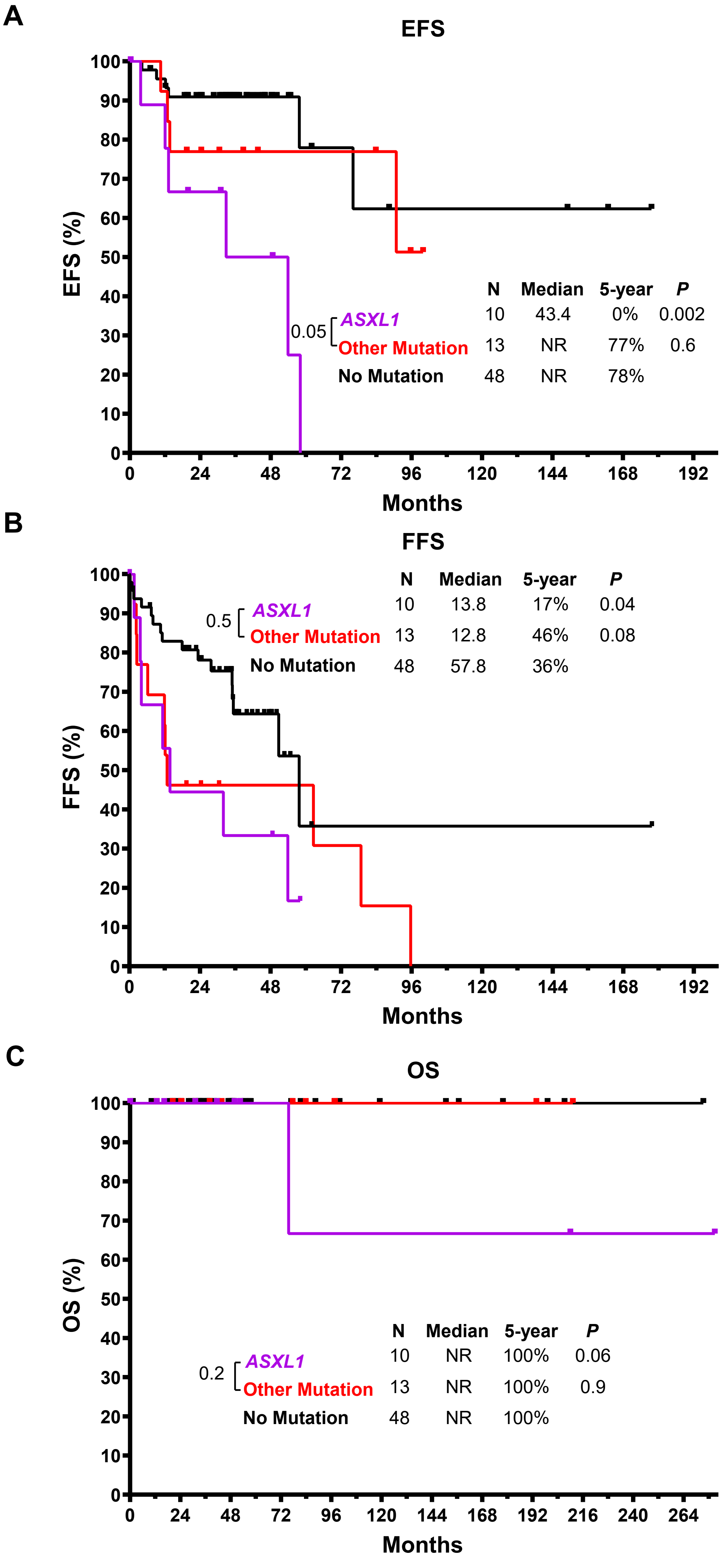

Supplemental Figure 4: Impact of *ASXL1* mutation detected at diagnosis on survival in chronic phase CML. A. Event-free survival (EFS) **B.** Failure-free survival (FFS) **C.** Overall survival (OS)

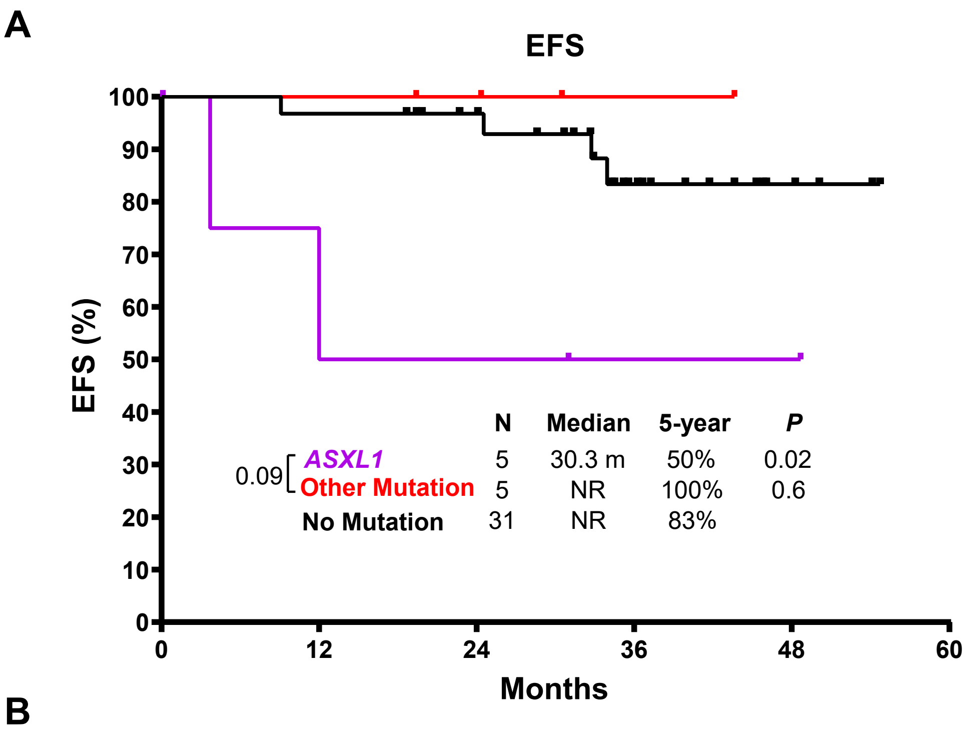

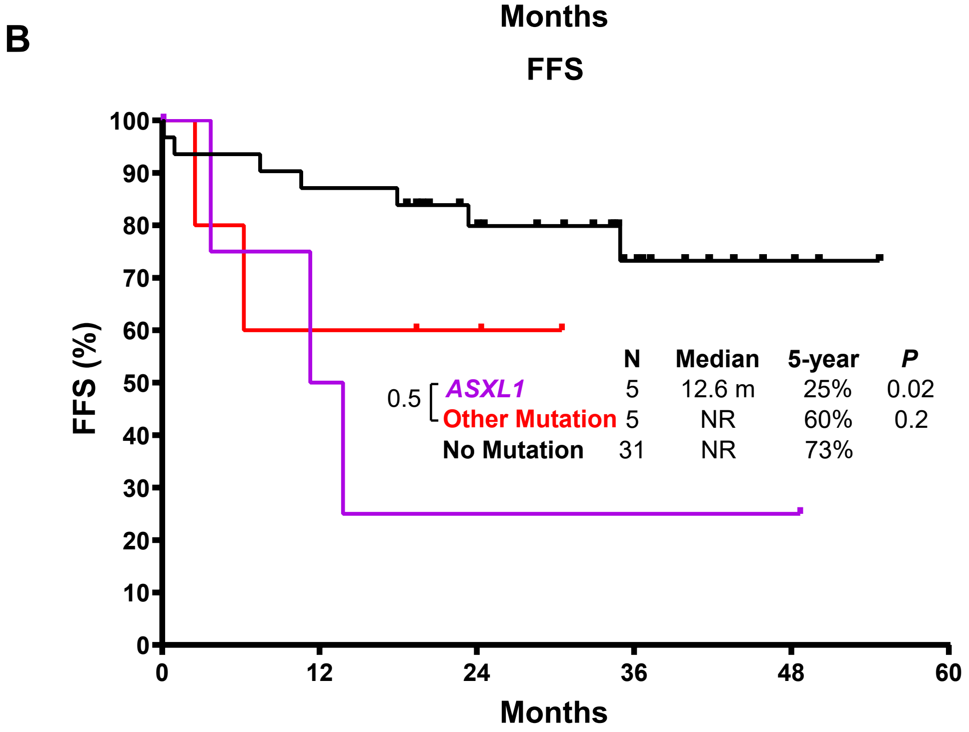

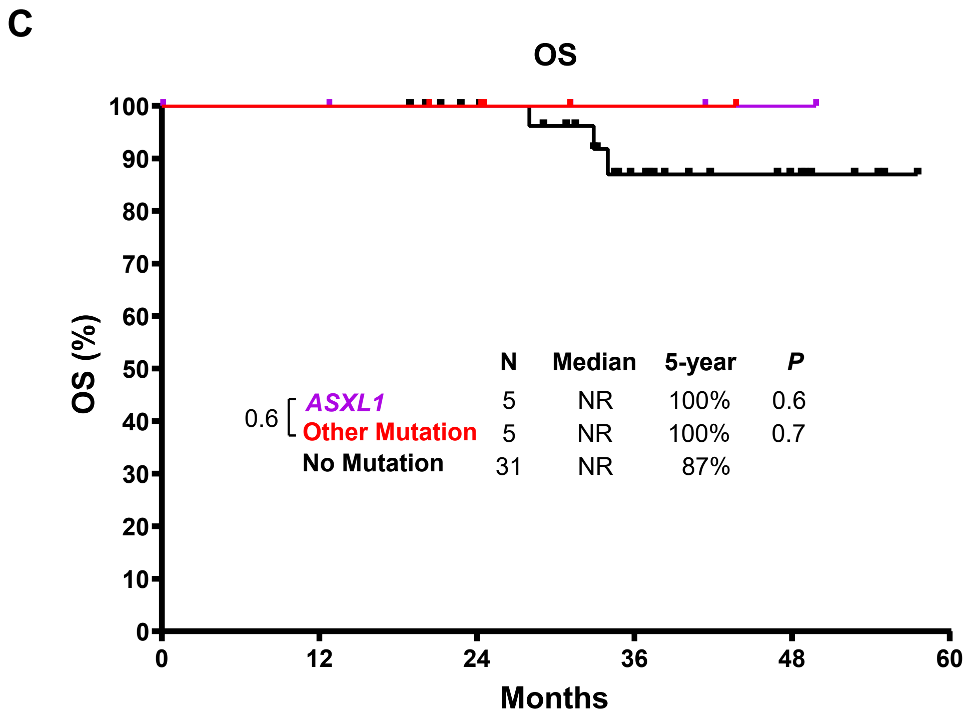

Supplemental Figure 5: Impact of *ASXL1* mutations detected at resistance/loss of response on survival in chronic phase CML. A. Event-free survival (EFS) **B.** Failure-free survival (FFS) and **C.** Overall survival (OS)

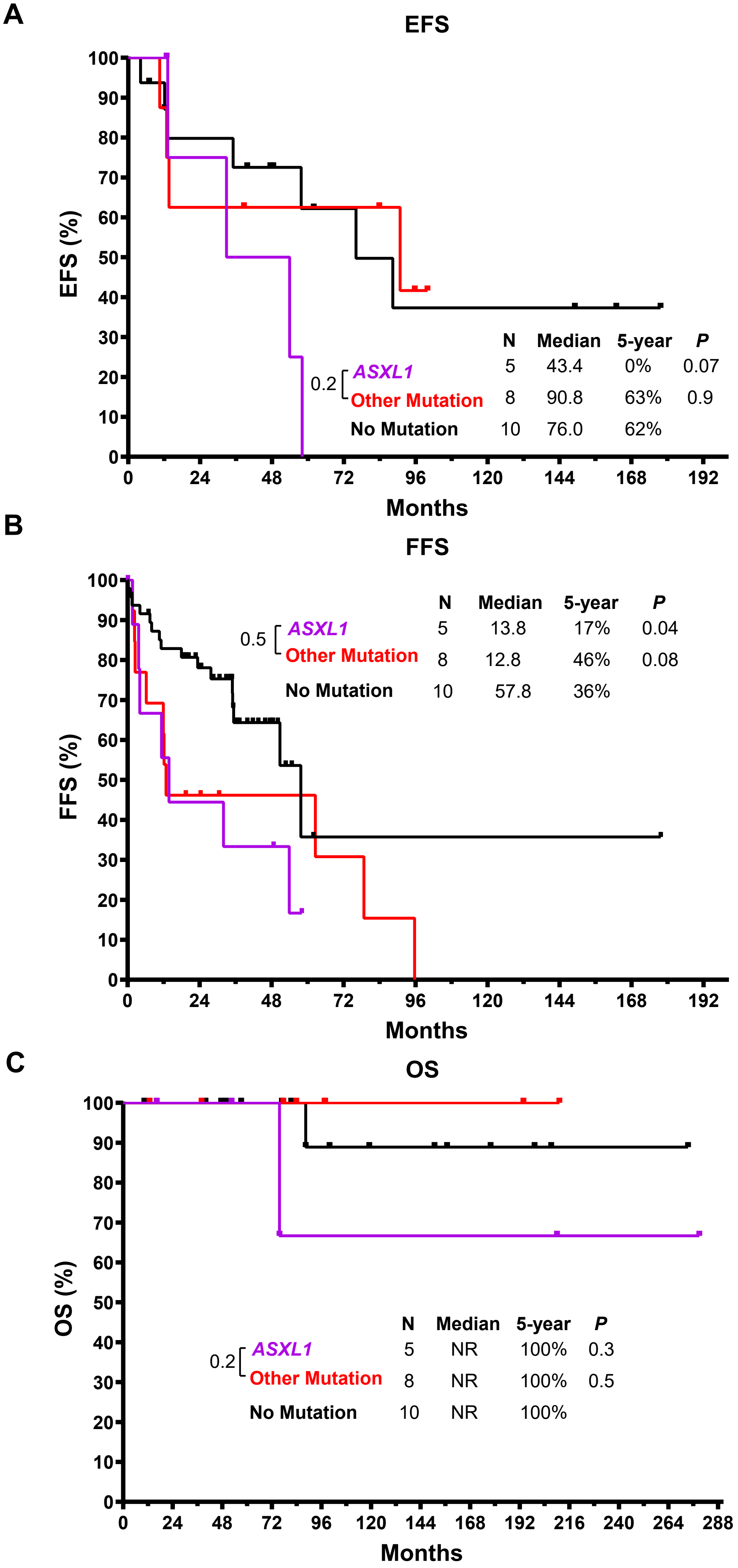

Supplemental Figure 6: Impact of non-*ABL1* gene mutations on survival in blast phase CML.
A. Event-free survival (EFS) **B.** Failure-free survival (FFS) **C.** Overall survival (OS)

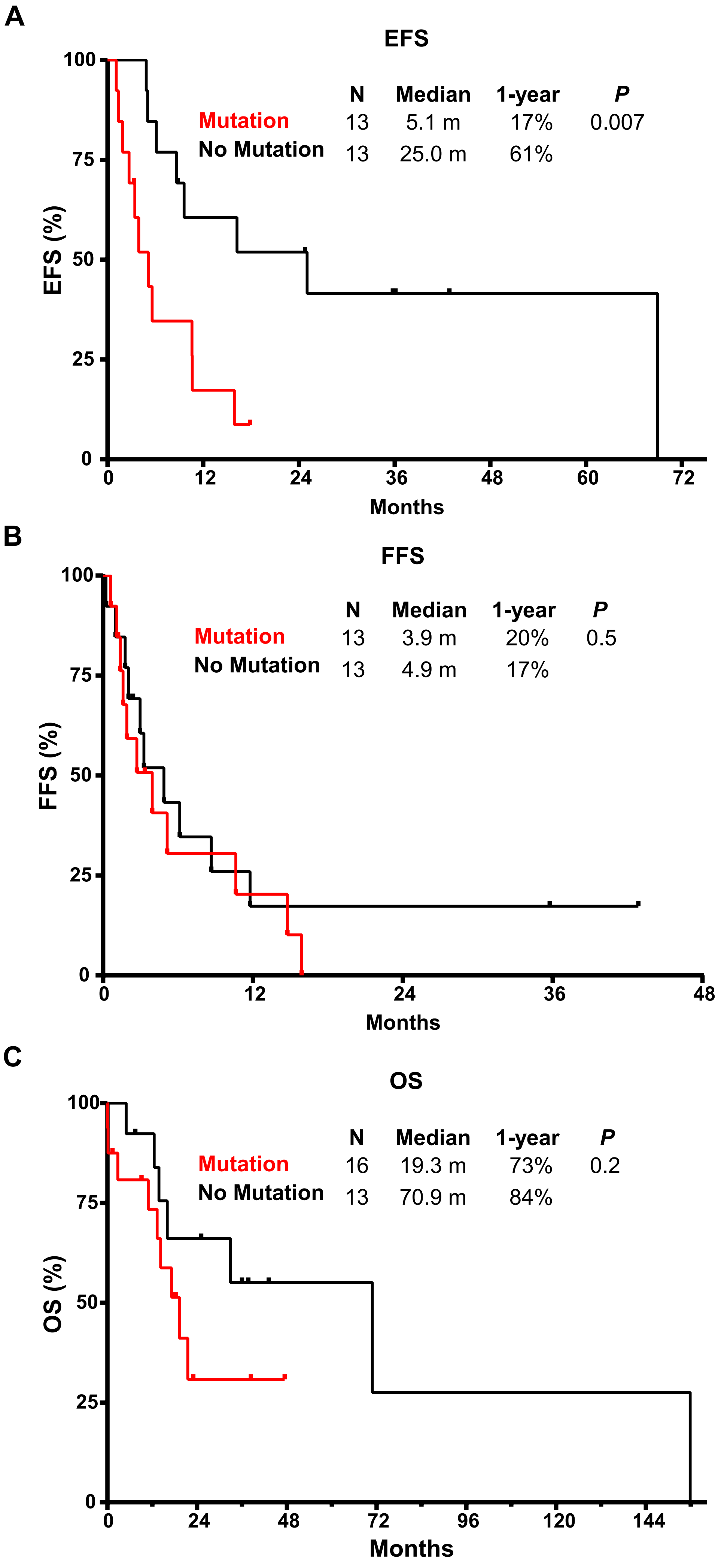

Supplement: Supplementary file 1 — Supplemental material [file 41408_2022_742_MOESM1_ESM.pdf]
